# Mildred T. Stahlman, MD—Neonatal trailblazer lived simple life in Tennessee, devoted to friends, colleagues, and patients

**DOI:** 10.1038/s41390-024-03762-8

**Published:** 2024-12-09

**Authors:** Corey Nason Reese, Jeff Reese, Jeffrey A. Whitsett

**Affiliations:** 1 CNR Communications, Brentwood, TN USA; 2 Department of Pediatrics, Vanderbilt University Medical Center, Nashville, TN USA; 3 Department of Pediatrics, University of Cincinnati, Cincinnati, OH USA

**Figure:**
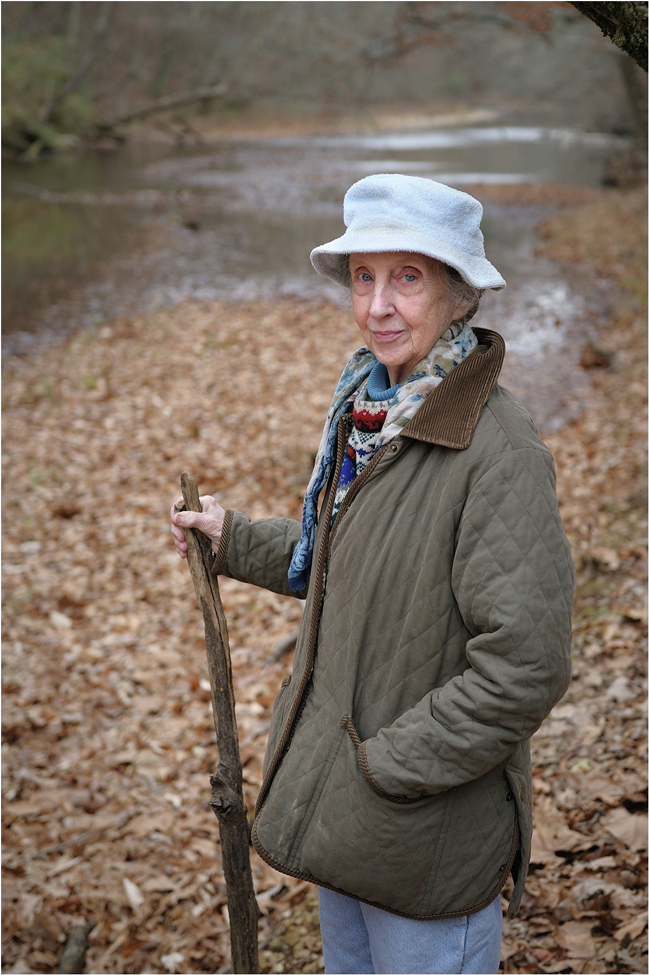
Mildred Stahlman, MD, stands in front of Hurricane Creek, which runs through her much-loved farm in Humphreys County, near Bucksnort, TN. Photographer: Kraig Haver

*Mildred T. Stahlman, MD*, died from natural causes on June 29, 2024, at the age of 101. **“**Millie” was born in 1922 to the prominent Stahlman publishing family in Nashville, Tennessee. Her father, James Geddes Stahlman (1893–1976), was the third-generation publisher of the Nashville Banner daily newspaper and a Vanderbilt alumnus, as was her sister, Ann Stahlman Hill (1921–2014). Her mother, Mildred  P. Thornton Stahlman Rhett (1895–1987), was a quintessentially southern woman with deep regional roots, providing the Stahlman daughters a rich environment steeped in Vanderbilt traditions. Due to World War II, Dr. Stahlman was admitted to an accelerated six-year program, graduating from Vanderbilt University in 1943 and Vanderbilt Medical School in 1946, where she received Phi Beta Kappa and Alpha Omega Alpha honors, respectively. Yet, while she was raised in the southern United States, she determinedly went beyond the conventions of her era and trailblazed new paths for women and minorities in medicine at every juncture of her career.

Millie Stahlman orbited in two distinct universes, having been selected by Vanderbilt for a unique exchange program, training in Sweden at the Karolinska Institute of Medicine in cutting-edge aspects of cardiorespiratory physiology (1949–1950) while simultaneously pursuing a career in medicine. Prior to her research fellowship in Sweden, she was one of four women in her medical school class and the sole woman in her clinical medicine internship at Lakeside Hospital in Cleveland (1946–1947). She completed her pediatric residency years at Boston Children’s Hospital (1947–1948), and Vanderbilt University Hospital (1948–1949), with an additional cardiology residency at La Rabida Sanitarium (1951, later Children’s Hospital) in Chicago. She collaborated with and trained clinicians and investigators throughout the U.S. and Europe. She founded the first sophisticated neonatal intensive care unit in the U.S. at Vanderbilt University, distinguished by her use of mechanical ventilation and invasive cardiorespiratory and blood gas monitoring, combined with intravenous fluid support, nursery hygiene, and dedicated intensive care space and personnel.^1,2^

Dr. Stahlman’s young pediatric career took a fortuitous turn when she was invited to join the laboratory of Professor Elliott Newman, MD, (1914–1973) Vanderbilt’s Director of the General Clinical Research Center, where he supervised a program of clinical physiology and research. Her work would result in a National Institutes of Health grant in 1954 to study transitional circulation and neonatal cardiorespiratory disease in her sheep lab. Newman encouraged her to apply for a larger grant in 1959, which enabled her to establish the innovative Vanderbilt Neonatal Intensive Care Unit with her physiology research laboratory uniquely located within the dedicated nursery space. In 1961, she enrolled her first infant that was diagnosed with pre-morbid respiratory distress and placed the baby in a modified iron lung, utilized negative and positive pressure mechanical ventilation, monitored blood gas values via umbilical lines, and remained at the bedside for 5 days.^1,3^

The recovery of that infant was a pivotal milestone in neonatal intensive care and transformed the practice of neonatology. Her survival also marked the beginning of Dr. Stahlman’s career as the director of the Vanderbilt NICU for the next 25 years. During that time, she traveled the U.S. and internationally—sharing her data, experiences, and techniques while learning from others. Her influence was felt through the transitional years of modern fetal and newborn physiology, to the birth of newborn intensive care, the early use of ventilators to support preterm infants, and the application of cell and molecular biology in the study lung formation and disease.

She created one of the first neonatal fellowship training programs (1961)^4,5^ and mentored more than 80 fellows from 20 countries, established one of the earliest regional transport systems in the U.S. in 1974,^6^ served on the committee to write the initial neonatal sub-board exams in 1975,^5^ and helped found or was an early member in perinatal societies, both national and abroad. She took a personal interest in the ethics of perinatology and gave thoughtful and challenging lectures on broad ethical considerations of neonatology and beyond, with her American Pediatric Society John Howland Award (1996) acceptance article, entitled, “Who Will Save Our Children?”^7^ Her final decades were spent in collaboration with Vanderbilt Professor Mary Phillips Gray, PhD, conducting lung morphology and immunohistochemistry studies as well as becoming an expert in the use of electron microscopy to study neonatal lung disorders. This sparked a 20-year collaboration with the Whitsett laboratory that combined Dr. Stahlman’s expertise in lung morphology with their novel transgenic and translational surfactant models.

Dr. Stahlman received numerous awards and distinctions in addition to the Howland Award including election to the Institute of Medicine (1984) and receiving the American Academy of Pediatrics Virginia Apgar Award (1987). She was elected president of the Southern Society for Pediatric Research (1961—in its inaugural year) and president of the American Pediatric Society (1984–85). Dr. Stahlman was selected by Vanderbilt Medical Alumni Association as a Distinguished Alumni (2002) and the seventh Distinguished Alumni by Vanderbilt University (2004). Internationally, she received the Swedish Medical Association Medal (1982) and membership in The Royal Swedish Academy of Sciences (1989).

She was a tiny woman with a heart of gold, the courage of a lioness, and a legendary temper if details in her NICU were overlooked. She personified the physician-scientist and was also a tireless advocate for child health, understanding and articulating the most fundamental issues governing infant well-being—including poverty, racism, access to or knowledge of health care systems, and pre-pregnancy health. For many of us, her unrelenting energy and commitment to the care of newborns served as a guiding inspiration for our new field—neonatology—as well as for women in science and medicine.

Yet, beyond her awards and single-minded determination to improve a patient’s care, which could sometimes lead to challenging teaching rounds in her NICU,^4^ what Dr. Stahlman will also be remembered for was that she was a great friend to many, with her influence spanning the globe for more than half a century.

Her life away from the NICU featured a restored log cabin at her country home in the Nashville suburb of Brentwood and two more cabins near an old farm in rural Bucksnort, TN. Guests invited to her farm would drive along a gravel road by Hurricane Creek, which ran through her property, toward the cabins and stable with five nearly feral Arabian horses, and a cold artesian spring where she kept her watermelons for an afternoon snack. Whitsett family members, as well as numerous other guests, would be greeted by a pack of dogs, many of whom Dr. Stahlman would have rescued or might have been dropped off at her home, ride horseback all morning for miles along the creek, with lunch eaten beside its banks. Children of friends, trainees, and colleagues—along with the adults—would swim in the clear creek water filled with trout. Virginia bluebells that bloomed in the spring were also a part of where she would refresh and recharge.

She loved Middle Tennessee, the culture, and its people—and was quick to share it with others. At the end of her life in her beloved suburban cabin, she enjoyed the company of one of her many dogs named “Queenie” and with a few close friends and family, she reflected on the beauty that surrounded her home.

Dr. Stahlman wouldn’t accept adulation and honors without pointing to those who came before her and liked to say that if she hadn’t done all this, someone else would have. However, we knew she was a remarkable woman who despite living in Nashville, which was a relatively small town in 1961, had the intellect and persistence to pioneer a specialized field of pediatrics that would enable herself and others to save countless babies around the world.

Mildred Stahlman was simply “Millie” to many of us. But she was also the treasured Dr. Stahlman to her patients and community, her institution, coworkers, and trainees who were also so fortunate to have traveled her long and distinguished path beside her.
